# Epigenetic Variability in the Genetically Uniform Forest Tree Species *Pinus pinea* L

**DOI:** 10.1371/journal.pone.0103145

**Published:** 2014-08-01

**Authors:** Enrique Sáez-Laguna, María-Ángeles Guevara, Luis-Manuel Díaz, David Sánchez-Gómez, Carmen Collada, Ismael Aranda, María-Teresa Cervera

**Affiliations:** 1 Departamento de Ecología y Genética. Centro de Investigación Forestal (CIFOR), Instituto Nacional de Investigación y Tecnología Agraria y Alimentaria (INIA), Madrid, Spain; 2 Unidad Mixta de Genómica y Ecofisiología Forestal, Instituto Nacional de Investigación y Tecnología Agraria y Alimentaria (INIA)/Universidad Politécnica de Madrid (UPM), Madrid, Spain; 3 Departamento de Biotecnología, Escuela Técnica Superior de Ingenieros de Montes (ETSIM), Universidad Politécnica de Madrid (UPM), Madrid, Spain; Ben-Gurion University, Israel

## Abstract

There is an increasing interest in understanding the role of epigenetic variability in forest species and how it may contribute to their rapid adaptation to changing environments. In this study we have conducted a genome-wide analysis of cytosine methylation pattern in *Pinus pinea*, a species characterized by very low levels of genetic variation and a remarkable degree of phenotypic plasticity. DNA methylation profiles of different vegetatively propagated trees from representative natural Spanish populations of *P. pinea* were analyzed with the Methylation Sensitive Amplified Polymorphism (MSAP) technique. A high degree of cytosine methylation was detected (64.36% of all scored DNA fragments). Furthermore, high levels of epigenetic variation were observed among the studied individuals. This high epigenetic variation found in *P. pinea* contrasted with the lack of genetic variation based on Amplified Fragment Length Polymorphism (AFLP) data. In this manner, variable epigenetic markers clearly discriminate individuals and differentiates two well represented populations while the lack of genetic variation revealed with the AFLP markers fail to differentiate at both, individual or population levels. In addition, the use of different replicated trees allowed identifying common polymorphic methylation sensitive MSAP markers among replicates of a given propagated tree. This set of MSAPs allowed discrimination of the 70% of the analyzed trees.

## Introduction

DNA Cytosine methylation has been shown to play a determinant role in a variety of molecular processes such as regulation of plant gene expression during development [1], imprinting [2] or genome stability including mobile elements control [3], [4] and polyploidization events [5], [6].

These functions have important implications not only in fields like developmental biology [1] but also in ecology and evolution. Epigenetic mechanisms have been proposed to contribute to adaptation in plants [7]–[9]. Several recent studies have identified correlations between epigenetic variability and adaptive population differentiation of plants in response to environmental stresses such as drought [10], [11], salinity [12], [13], or damage by herbivores [14], [15].

Environmentally-induced epigenetic changes have been shown to mediate phenotypic plasticity by regulation of specific gene expression as well as plant development after a change in environmental conditions [16]–[18]. It is also known that epigenetic variability can be independent from genetic variability [19]–[22], becoming a source for adaptive potential in itself [18], [23]–[25]. Epigenetic changes induced by stress are potentially reversible but some modifications are not only inherited from cell to cell during mitosis but they can also be inherited across generations [26]–[29]. This so-called “stress memory” allows plants to retain active molecular mechanisms after the stress signal disappears, thus responding more efficiently to recurrent stressful conditions [16]–[18]. Stress memory can considerably increase the adaptive potential and may help plants to cope with changing environmental conditions [24].

Although the number of studies about epigenetic variation associated with biotic and abiotic stresses in plants is increasing, few studies are focused on forest tree species, with perhaps the exception of poplar [10], [11], [30], [31]. Trees, and especially conifers, are key models for the study of stress adaptation due to their longevity and long-life cycles [9], [32]. Some conifers like *Sequoia sempervirens* or *Pinus longaeva* can live for 3,000 and 5,000 years, respectively [33]. Therefore, these species must cope with very variable environments through their life spans. Conifer genomes are very large, with genome sizes ranging between 20,000 and 30,000 Mbp, which on average is 200-fold larger than Arabidopsis genome and 6–10-fold larger than the human genome [34], [35]. Thus, regulatory machinery for gene expression and genome stability must be a key factor for these species in order to survive under changing environmental conditions. From an ecological and economic standpoint, conifers are the most important group of gymnosperms. Altogether, they represent 39% of the world's forest area [36]. However, little research about epigenetics, and more specifically cytosine methylation, has been done in conifers. The most explored fields in conifers have focused on epigenetic processes in tree development [37]–[39] and epigenetic memory to environmental factors [40]–[42].

*Pinus pinea* L. (stone pine) is one of the most ecologically, economically and socially important Mediterranean forest tree species. It is patchily distributed in the North and Southeast Mediterranean area, from Portugal to Syria. Stone pine is characterized by a very low genetic variation [43]–[45] and high adaptive plasticity that increases its global fitness [46]–[49]. High degree of phenotypic plasticity has been found in response to water availability. The analysis of propagated trees grown under water deficit revealed a significant variation in functional traits [50]. This genetically depauperated but plastic species constitutes an optimal system to study natural epigenetic variability and its potential to shape phenotypic plasticity [24].

The main goal of this work was to analyze cytosine methylation in *Pinus pinea* genome. Despite the lack of genetic variation of this species we expect to identify methylation variability between individuals that might explain the significant variation in functional traits observed in the species. Two different objectives were outlined: 1) Analyze if *P. pinea* genome is methylated and the extent of methylation. 2) Analyze if cytosine methylation is correlated with genetic variability or if cytosine methylation patterns differ among and within individuals. To carry out this study we have analyzed DNA from vegetatively propagated individuals from natural populations of stone pine using two genome wide profiling techniques, Amplified Fragment Length Polymorphism (AFLP) and Methlylation Sensitive Amplified Polymorphism (MSAP) surveying both genetic and epigenetic variability, respectively.

## Materials and Methods

### Plant Material

A total of 20 one-year-old seed-grown individuals from five natural populations representing the distribution of *Pinus pinea* L. in Spain (Tordesillas, Bogarra, Biar, Doñana and Palafrugell; Table S1 and Figure S1) were selected for this study. The two most represented populations, Tordesillas and Bogarra, have contrasting climates; Tordesillas has a colder continental climate while Bogarra has a temperate Mediterranean climate. Vegetative propagation of these individuals was conducted by planting cuttings in a mix of equal amounts of peat and river sand using 1% IBA (Rhizopon AA powder) to promote rooting. A set of 95 rooted cuttings (ramets) were obtained. After two months, the ramets were transplanted into 1.2 l containers with a 3∶1 mixture of peat and river sand. Plants were grown in a climatic chamber under controlled conditions (photosynthetic photon flux density (PPFD) of 600–650 µmol m^−2^ s^−1^, temperature of 20°C, relative humidity of 60% and photoperiod of 16/8) placed in a random block design consisting in four blocks with 1–2 ramets of each propagated tree per block. Four months later, needles of similar developmental stage and 2 cm below tip of main apical shoots were collected from every ramet and stored at −80°C for subsequent DNA extraction. Elongating shoots with very young needles were discarded during sampling as well as needles from initial rooted cuttings, which originated from the mother tree. In this respect, needles of same ontogenic state were carefully selected to reduce methylation variation associated to different developmental stages.

### DNA extraction and quantification

DNA was extracted from needles grinded in a mixer mill (Retsch MM300) using Dellaporta's protocol [51] modified as described in Cervera et al 2005 [52] Extracted DNAs were quantified using a spectrophotometer (Thermo Scientific, Nanodrop 1000). DNA integrity was determined by agarose gel electrophoresis (1% agarose; 1x TBE; 0.03 µg/ml EtBr).

### AFLP analysis

A total of 59 ramets from the 13 propagated trees belonging to the two most represented Spanish populations were analyzed using Amplified Fragment Length Polymorphism (AFLP) [53]. This analysis was performed by digesting 500 ng DNA with *Eco*RI/*Mse*I restriction enzymes according to Cervera et al. [54]. The number of selective nucleotides for the two consecutive amplification steps was *Eco*RI + 1/*Mse*I +1 for the pre-amplification and *Eco*RI +3/*Mse*I +3 for selective amplifications. Two primer combinations (Table S2) were used: *Eco*RI + ACC/*Mse*I + CCA and *Eco*RI + ACG/*Mse*I + CCA. *Eco*RI +3 selective primers were labeled at their 5′ ends with fluorescence dye 800 IRDye to allow visualization of the fragments on a Li-Cor 4300 DNA Analyzer (Li-Cor Biosciences, Lincoln, NE).

AFLP amplified products were separated by electrophoresis in 25 cm denaturing polyacrylamide gels [16% Long Ranger 50% Gel Solution (Lonza), 7 M urea, 1x TBE], run at 1500 V and 45°C. Before loading, samples were denatured by adding an equal volume of formamide buffer (98% formamide, 10 mM EDTA, pH 8.0, and 0.06% bromophenol blue) and heated for 2 minutes at 94°C.

Scoring of the resulting fragment patterns was based on a presence/absence (1/0) approach. Only markers with an undoubtedly reliable score of at least 95% of the samples (less than 5% of missing data) were considered to estimate genetic variability.

### MSAP analysis

Methylation Sensitive Amplified Polymorphism technique [55], modified by Cervera *et al*. [20], was used to analyze DNA cytosine methylation level and pattern in the genome of *P. pinea* propagated trees.

Due to the large genome size of conifers [35] several steps of the technique were optimized for pine species. The initial amount of DNA digested with the two restriction enzyme combinations (*Eco*RI/*Hpa*II and *Eco*RI/*Msp*I), was increased up to 500 ng [52]. A combination of *Eco*RI +1//*Hpa*II/*Msp*I +1 selective nucleotides in the pre-amplification followed by *Eco*RI +3//*Hpa*II/*Msp*I +3 selective nucleotides in the selective amplification step provided the best results. *Eco*RI +3 selective primers were labeled with fluorescent dye as in AFLP.

Initially, nine different *Eco*RI +3//*Hpa*II/*Msp*I +3 primer combinations (AAC/AAT, ACA/AAT, ACT/AAT, ATC/AAT, AAC/ACT, ACA/ACT, ACG/ACT, ATC/ACT, AAC/ATC; Table S2) were tested on a sample subset to identify the most informative combinations. For this purpose, each propagated tree was represented by a pool made of equimolar amounts of pre-amplified DNAs from its corresponding ramets. All pools were analyzed using the nine primer combinations to compare their MSAP profiles, selecting the two most informative ones: *Eco*RI + AAC//*Hpa*II/*Msp*I + AAT (AAC/AAT) and *Eco*RI + ACA//*Hpa*II/*Msp*I + AAT (ACA/AAT). These two primer combinations were used to analyze cytosine methylation patterns of the 95 ramets individually. Electrophoresis settings were similar to those applied for AFLP analysis.

### Scoring and interpretation of MSAP fragment patterns

Comparative analysis between *Eco*RI/*Hpa*II and *Eco*RI/*Msp*I profiles for each primer combination allows establishing the methylation status of each targeted restriction site. Methylation-sensitive endonucleases *Hpa*II and *Msp*I cleave CCGG sequences with differential sensitivity to methylation at the inner or outer cytosine: *Hpa*II does not cut if one or both cytosines are full-methylated (methylation occurs in both DNA strands) but cleaves when cytosine methylation occurs in a single strand. *Msp*I does not cut if the outer cytosine is methylated in one or both strands [56], [57].

Initially, separated matrices were constructed for *Eco*RI/*Hpa*II and *Eco*RI/*Msp*I fingerprints. MSAP fragment presence or absence was visually determined by two independent observations. We detected fragments differing in intensity probably due to different degree of cytosine methylation in different cell types of the analyzed samples. Only markers with an undoubtedly reliable score of at least 95% of the samples (less than 5% of missing data) were considered to estimate epigenetic variability. Rationale for the comparative scoring was based on differential presence/absence of a particular fragment in *Hpa*II and *Msp*I digestions. Thus, for a given sample, hypomethylation (fragment present in *Eco*RI/*Hpa*II and *Eco*RI/*Msp*I fingerprints) and full methylation of both cytosines (fragment absent in *Eco*RI/*Hpa*II and *Eco*RI/*Msp*I fingerprints relative to other samples) were coded as 0. Since *P. pinea* shows undetectable levels of genetic variation, the loss of the target sequence motif cannot be considered within this class. On the other hand, for a given sample, full methylation of the internal cytosine (fragment only present in *Eco*RI/*Msp*I fingerprint) and hemi-methylation of the external cytosine (fragment only present in *Eco*RI/*Hpa*II fingerprint) were codified as 1. The resulting integrated matrix was used for statistical analysis.

**Figure 1:**
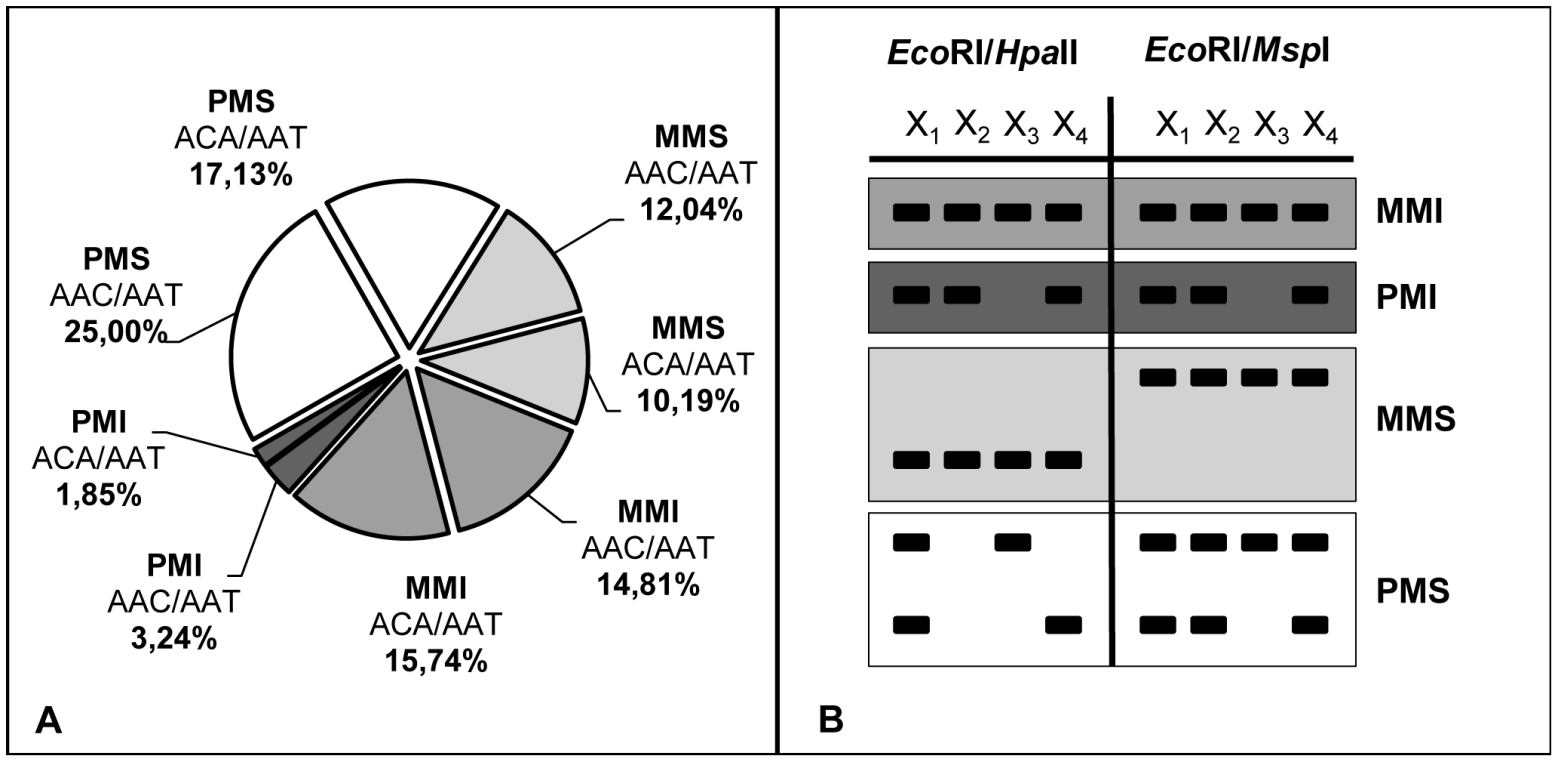
*Pinus pinea* genome-wide methylation analysis based on MSAPs. Four different classes of MSAPs were identified depending on their cytosine methylation status and their polymorphic profile: Monomorphic Methylation Insensitive (MMI), Polymorphic Methylation Insensitive (PMI), Monomorphic Methylation Sensitive (MMS) and Polymorphic Methylation Sensitive (PMS). a): percentage of MSAP markers assigned to each class; b) fragment pattern associated with each class.

MSAP markers were then classified according to their global pattern in all samples (Figure 1-B). Two main groups were identified depending on whether there was at least a difference between *Eco*RI/*Hpa*II and *Eco*RI/*Msp*I digestions profiles. Markers were then identified as Methylation Insensitive (MI) when no difference was found between the two digestions profiles for any sample and as Methylation Sensitive (MS) when difference between both profiles was found for one or more samples. These two groups were further split according to whether difference among samples was found. Following this reasoning, MI markers presenting the same pattern among all samples were classified as Monomorphic Methylation Insensitive (MMI) markers and MIs presenting differences among samples were classified as Polymorphic Methylation Insensitive (PMI) fragments. MS fragments were classified as well into Monomorphic Methylation Sensitive (MMS), when they showed different pattern between isoschizomers but not among samples, and Polymorphic Methylation Sensitive (PMS) fragments when at least one sample did not show the same profile.

### Statistical analysis

Percentages of cytosine methylation were subjected to analysis of variance (ANOVA; Statistica [58]) to unveil differences in the degree of cytosine methylation among propagated trees. MSAP markers showing the same profile among all individuals derived from the same propagated tree were identified. To analyze its discriminative power, epigenetic similarity (ES) was estimated from the number of shared amplified fragments by using the Dice similarity coefficient [59] [ES(ij) = 2a/(2a+b+c)] where ES(ij) is the measure of ES between the individuals i and j, a is the number of polymorphic fragments that are shared by i and j, b is the number of fragments present in i and absent in j, and c is the number of fragments present in j and absent in i. The resultant matrix was subjected to cluster analysis by the unweighted pair-group method analysis (UPGMA) and a dendrogram was constructed according to the clustering. Clustering was subjected to bootstrapping in order to obtain values for the reliability of the consensus dendrogram. Similarity matrix was obtained using DistAFLP software [60]. Using Bootstrap Computation, 1000 matrices were obtained. Cluster analysis and dendrogram construction were performed with PHYLIP phylogeny software package (programs Neighbor and Consense, respectively) [61]. Dendrogram was visualized with MEGA software [62].

Analysis of the Molecular Variance (AMOVA) [63] based on polymorphic methylation sensitive markers was performed over the 59 ramets of the two most represented populations, Tordesillas and Bogarra (Arlequin, version 3.5 [64]). Locus by locus AMOVA was performed to identify markers with a significant effect on population differentiation or differentiation of propagated trees (Table S3). These markers were then used to perform a Principal Component Analysis (PCA; Statistica [58]).

## Results

### Genetic variability in *Pinus pinea*

A total of 59 ramets from 13 propagated trees of the two most represented Spanish populations (Tordesillas and Bogarra) were analyzed using Amplified Fragment Length Polymorphism (AFLP). A total of 215 AFLPs were identified with confident reliability using two primer combinations (*Eco*RI + ACC/*Mse*I + CCA and *Eco*RI + ACG/*Mse*I + CCA). A single AFLP fragment pattern was observed and no variation was found among ramets from each propagated tree as well as among different propagated trees.

### Epigenetic variability in *Pinus pinea*

DNA methylation variability among the 95 ramets from the 20 propagated individuals was analyzed comparing MSAP profiles. The two selected MSAP primer combinations yielded a total of 216 scored markers (Table S4) from which 139 were classified as MS and 77 as MI. Within MS markers, 91 were identified as PMS (42.13% of the total number of MSAPs). The remaining 48 MS markers were identified as MMS MSAPs. Out of the 77 MI markers, 66 were found to be MMI MSAPs. The remaining 11 MSAPs (5.09% of the total number of MSAPs) were identified as PMI. Ten out of these 11 PMI MSAPs showed a different pattern in at least one ramet of the propagated trees. Detailed classification per primer combination is shown in Figure 1-A.

The *Eco*RI + AAC//*Hpa*II/*Msp*I + AAT (AAC/AAT) primer combination was the most informative with 119 out of the 216 amplified MSAPs analyzed. The main difference between primer combinations was found in the number of PMS markers since MMS, MMI and PMI markers showed similar values for the two primer combinations (Figure 1).

**Figure 2:**
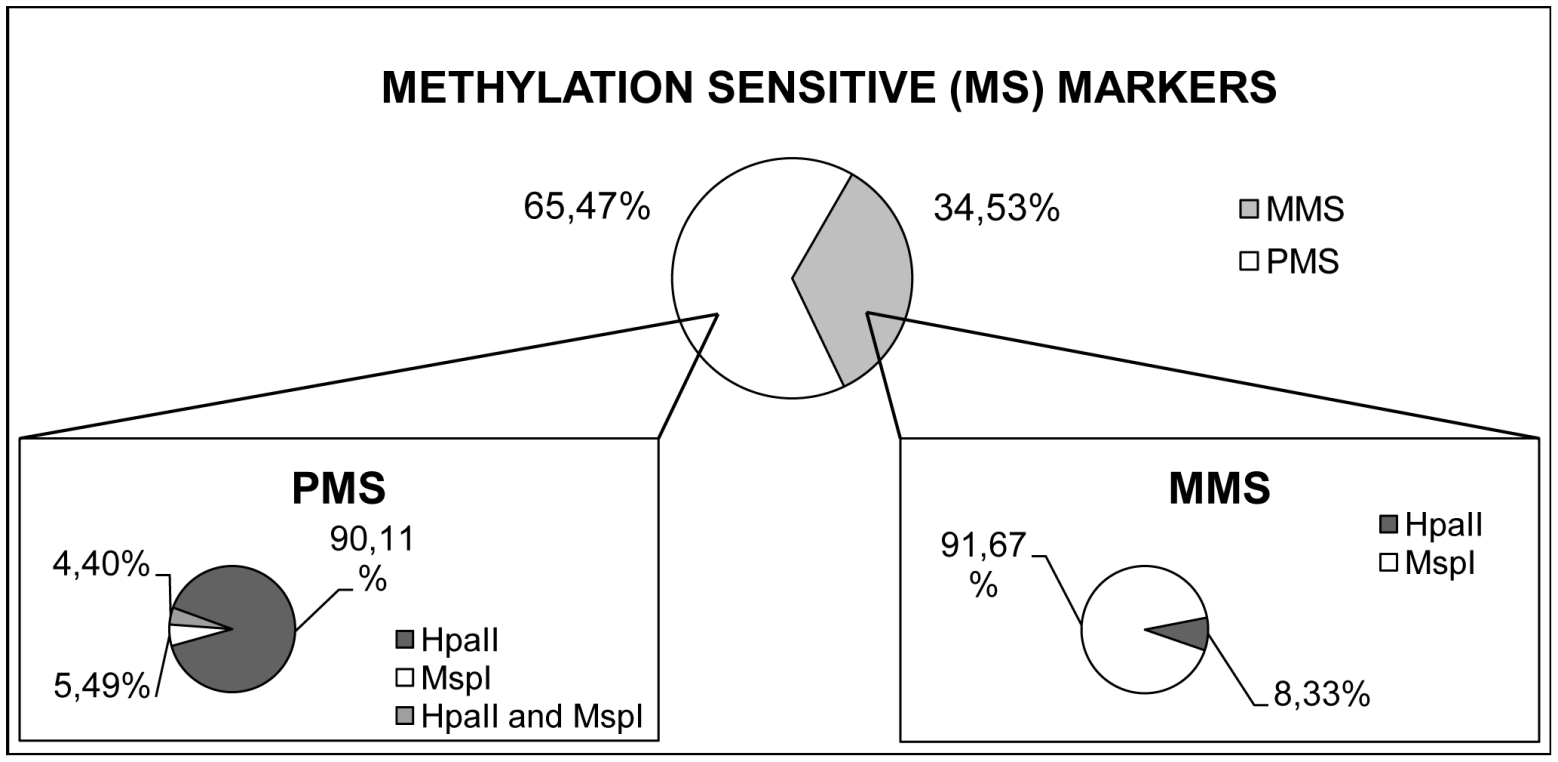
Detailed isoschizomer-based analysis of Methylation Sensitive fragments (MS). Comparison of Methylation Sensitive (MS) fragments between *Eco*RI/*Hpa*II and *Eco*RI/*Msp*I profiles. PMS.- Polymorphic Methylation Sensitive fragments; MMS.- Monomorphic Methylation Sensitive fragments.

Comparison of *Eco*RI/*Hpa*II and *Eco*RI/*Msp*I profiles showed contrasting levels of polymorphism. While *Eco*RI/*Msp*I provided a higher number of MSAPs than *Eco*RI/*Hpa*II, 116 versus 91, their fragment patterns were less polymorphic. In particular, 82 out of the 91 PMS markers showed variation only in the *Eco*RI/*Hpa*II profiles, 5 only in the *Eco*RI/*Msp*I profiles and the remaining 4 were associated with polymorphic MSAPs identified in both profiles (Figure 2). Thus, 91.67% of the MMS fragments were only found in *Eco*RI/*Msp*I profiles (Figure 2).

We explored differences in methylation level among the propagated trees estimating the number of MS markers detected for each ramet vs. the total number of MSAPs. The resulting mean value and standard deviation of all ramets corresponding to the same propagated tree were calculated. Values ranged from 42.73±0.88% (Pal 27) to 47.90±0.42% (Tor 27) (Table 1). DNA methylation significantly varied among the 20 different propagated trees (ANOVA, p<0.0001). Cytosine methylation polymorphism among ramets of each propagated tree, based on PMS detected among ramets vs. total number of MSAP fragments, was also calculated with values ranging between 0.46% (Tor 3) and 9.72% (Don 13).

**Table 1:** Quantification of cytosine methylation in all analyzed genotypes.

Propagated tree	DNA methylation (mean and std. dev. in %)	Number of PMS MSAPs
Tor - 3	44,97±0,37	1
Tor - 7	44,67±1,08	8
Tor - 12	44,48±0,81	4
Tor - 13	44,21±1,71	16
Tor - 24	44,47±0,46	12
Tor - 25	45,05±0,28	14
Tor - 27	47,9±0,42	6
Tor - 29	43,37±0,63	16
Bo - 13	46,54±0,83	3
Bo - 14	45,15±0,56	5
Bo - 18	43,7±0,96	10
Bo - 20	46,98±0,47	2
Bo - 21	46,55±1,09	16
Don - 10	45,69±0,34	8
Don - 13	46,15±1,05	21
Don - 15	45,26±1,79	17
Bi - 23	47,12±2,27	9
Bi - 37	44,21±0,85	20
Pal - 19	45,05±1,18	11
Pal - 27	42,73±0,88	9

Percentage of cytosine methylation and number of polymorphic fragments are provided for each genotype.

**Figure 3:**
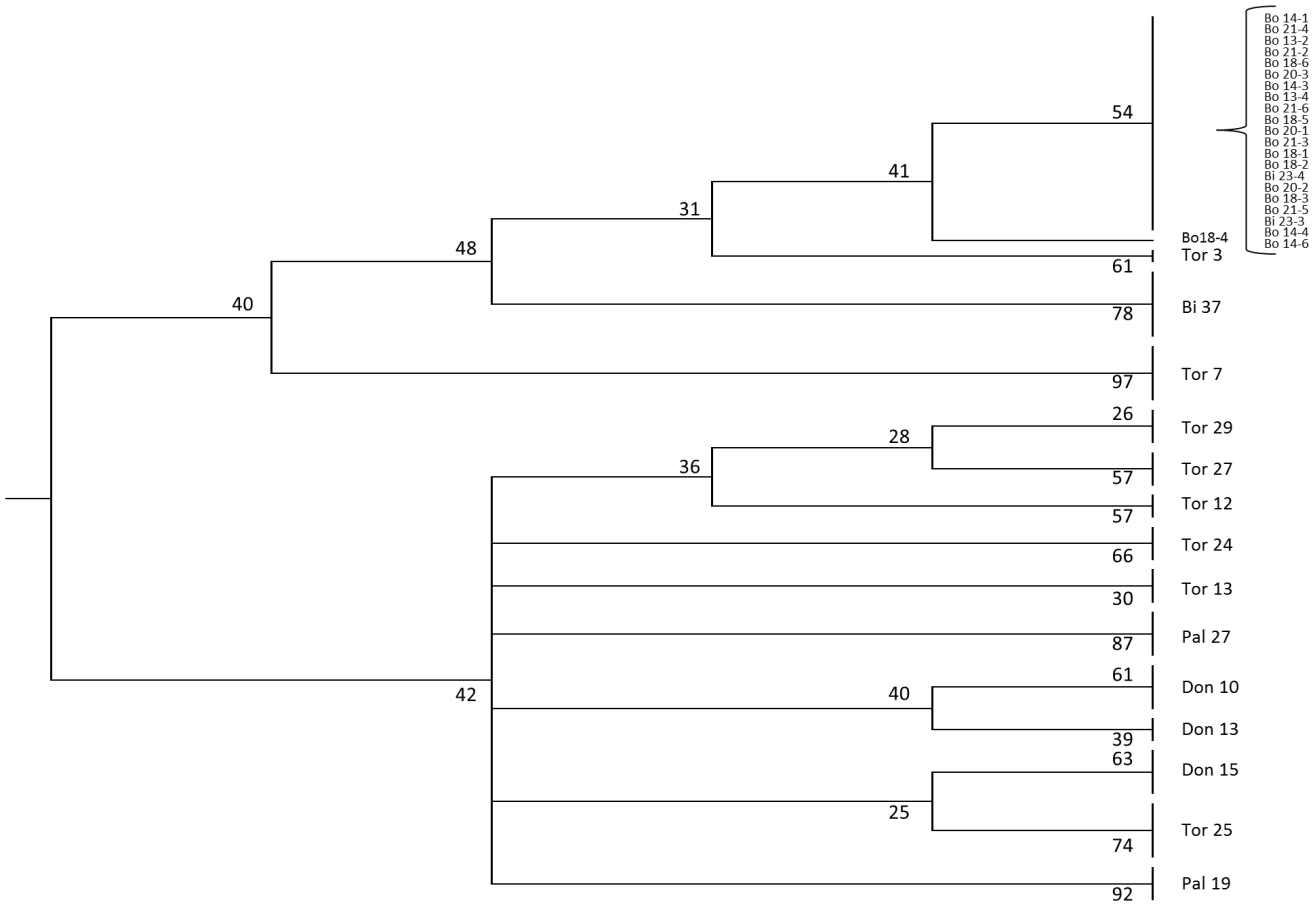
UPGM tree for genotype identification. Genetic similarity was calculated and bootstrapped UPGMA clustering was performed for genotype discrimination. Bootstrap computation percentages are shown over the different branches. Tree has been condensed a 25% and clones from the same genotype clustering together are labeled under the genotype code name for easier visualization.

Similarity analysis among MSAP profiles of the propagated trees allowed the identification of 15 PMS that, while being polymorphic among the analyzed trees, shown the same pattern among their vegetatively propagated ramets (Table S4). These markers were used to calculate an epigenetic similarity matrix based on DICE coefficient and to perform an UPGMA cluster analysis. As a result, the use of the 15 PMS MSAPs allowed to identify 14 out of the 20 studied individual trees (Figure 3) meaning that for these trees, all their ramets clustered together in common branches. Bootstrap values for these clusters were above 50% in 11 of the 14, and above 25% in all cases. All propagated trees from both Bogarra and Doñana populations were clustered as well as 5 out of 8 trees from Tordesillas. Trees from both Bihar and Palafrugell populations were not clustered together.

Although the number of analyzed trees is scarce to approach population epigenetic studies, we roughly estimated variability of DNA cytosine methylation associated with the studied populations. For this purpose, the mean value of the methylation levels obtained for all propagated trees of a given population was calculated. Palafrugell was the population whose individuals showed the lowest level of DNA methylation with 43.89±1.64% methylated cytosines, followed by Tordesillas, Biar, Doñana and Bogarra with 44.89±1.32%, 45.66±2.06%, 45.70±0.44% and 45.78±1.35%, respectively. We also found variation in the percentage of PMS MSAPs among populations (for a given population, PMS MSAPs vs total MSAPs). The less polymorphic population was Palafrugell (13.89%) and the most polymorphic one was Tordesillas (27.31%). Biar, Bogarra and Doñana showed intermediate values of 16.67%, 17.13% and 18.52%, respectively.

**Figure 4:**
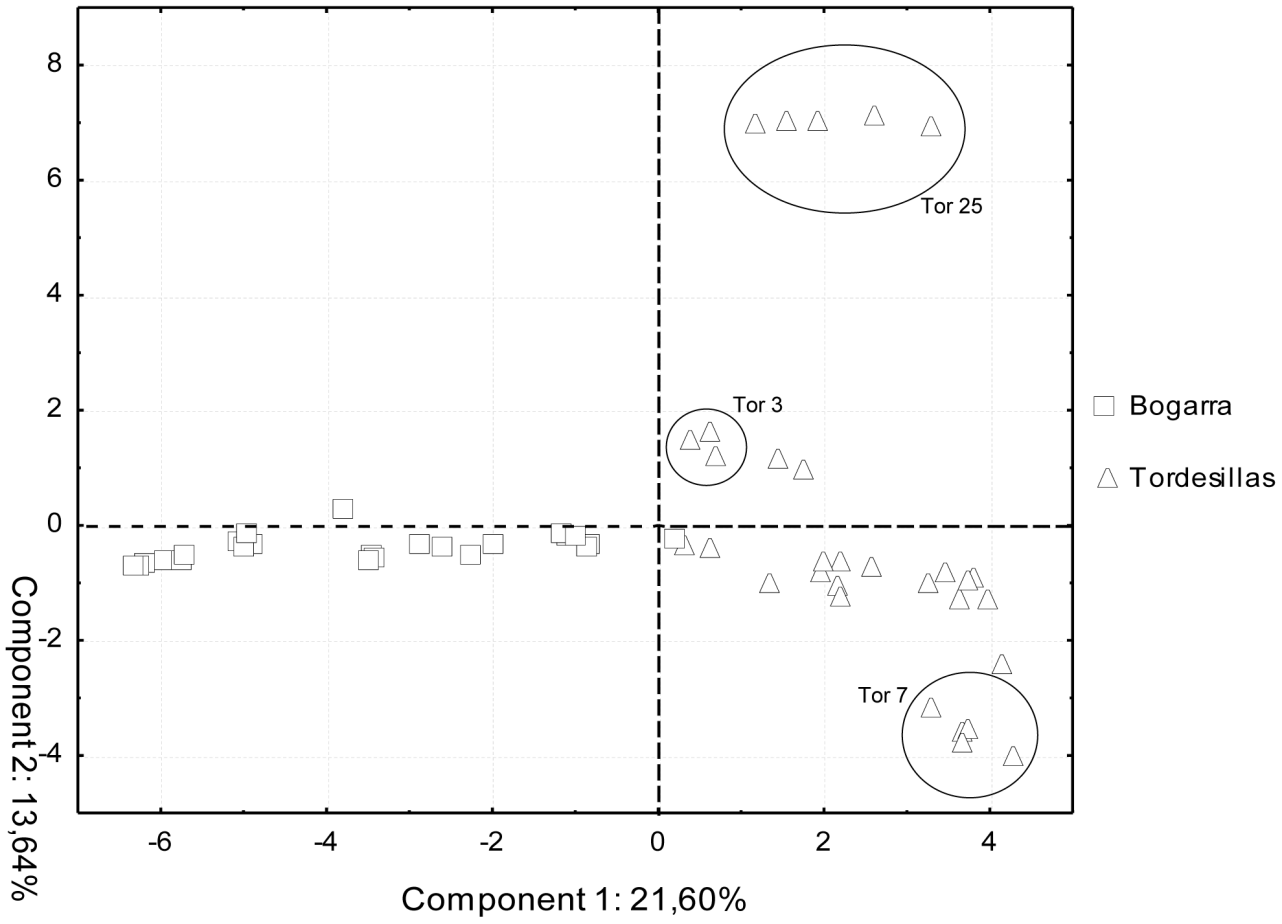
Two dimensional PCA scatter plot for population differentiation. Principal Components was performed to analyze samples belonging to Tordesillas and Bogarra populations using MSAPs with significant epigenetic effect differentiating populations and propagated trees. Bogarra ramets are identified by squares and Tordesillas ramets by triangles. Propagated trees which ramets clustered together are highlighted with circles. First component (X axis) gathers 21.60% of the variation and second component (Y axis) 13.64.

We tested the discriminative power of MSAP markers for the same two populations analyzed with AFLP technique by performing an Analysis of the Molecular Variance (AMOVA). The fixation index between populations (F_ST_) was 0.274 (p<0.0001). A locus-by-locus AMOVA was performed to determine which markers showed statistically significant epigenetic variation among populations. The resulting 52 markers (69.3% of the total polymorphic MSAPs identified in the two populations; Table S3) were used to differentiate populations and propagated trees using a Principal Components Analysis. First two components accounted for 35.24% of the total variance (comp. 1 = 21.60%; comp. 2 = 13.64%). Scores for these two components of each analyzed ramet were plotted in Figure 4. First component clearly differentiated both populations. In addition, it was possible to identify three propagated trees from Tordesillas population (Tor 3, Tor 7 and Tor 25) whose ramets clustered in separate groups.

## Discussion

Stone pine has been described as a genetically depauperated species, showing a very low level of genetic diversity [43]–[45]. The almost undetectable genetic variation has made it very difficult to genetically distinguish stone pine trees. In this study, we were unable to identify a single polymorphic marker using the multi-loci technique AFLP, making impossible genetic discrimination of the analyzed trees. This lack of genetic variation together with the relevant phenotypic plasticity displayed by this species [50], supports *Pinus pinea* as a suitable model for studying epigenetics and its ecological and evolutionary implications [24].

In this study we have analyzed DNA cytosine methylation patterns of 20 selected *Pinus pinea* individuals from natural populations covering the area of distribution of the species in Spain, as a first attempt to characterize this species. Due to the limited number of cDNAs that are publicly available for this non-model tree species, MSAP technology was used to analyze the methylation status of anonymous cytosines in *P. pinea* genome. We have detected that 64.36% of the analyzed cytosines at CCGG motifs were methylated. The observed percentage of stone pine genome-wide cytosine methylated sites was at least 10% higher than those reported for both annual and other perennial plants [6], [12], [21], [65]–[67]. This result is in agreement with genome hypermethylation of conifer genomes proposed by Nystedt et al. [68] as one of the mechanisms underlying conifer genome evolution. It is interesting to mention that most of the markers that were not selected for the analysis (DNA fragments undoubtedly scored in less than 95% of the analyzed samples) were Methylation Sensitive. Additionally, the degree of cytosine methylation is expected to be even higher since fully methylated sites cannot be detected with the MSAP technique [21], [66]. The broad presence of DNA methylation found in *Pinus pinea* genome might be related to certain extent with the repetitive nature of conifer genomes [68]. DNA methylation is known to control activity of mobile elements, protecting plant genomes against their mobilization [4], [69] and transposable elements are abundant in conifer genomes. Pseudogenes, which are also very abundant in conifers, have been described in mammal genomes as elements encoding long noncoding RNAs involved in the epigenetic regulation of gene expression [70].

This study has also revealed a high level of PMS markers meaning that 42.13% of the total MSAPs analyzed (or 65.46% of MS markers) showed cytosine methylation variation. Taking into account that this study comprises ramets belonging to 20 trees from five populations, this result becomes especially significant to picture the cytosine methylation landscape of the species. Several studies suggest that cytosine methylation variability in particular, and epigenetic variability in general, may be associated with phenotypic plasticity in traits with potential for improving local adaptation [12], [18], [65]. It has been shown in *Arabidopsis thaliana* that epigenetic diversity favors functional diversity associated with productivity and stability of populations [71]. The epigenetic variation found in *Pinus pinea* may play a role in the fitness of the species by acting as an alternative source of variability, different to genetic diversity, with evolutionary consequences.

Better understanding of this high DNA methylation variation can be achieved by comparing isoschizomers profiles associated to *Eco*RI/*Hpa*II and *Eco*RI/*Msp*I digestions. Most of the cytosine methylation variation detected (90% of the PMS fragments) was associated with *Eco*RI/*Hpa*II profiles, as the result of fragment detection due to *Hpa*II digestion of hemimethylated external cytosines at the CCGG sites (^m^CNG), that other samples lack because of fully methylation of the inner (C^m^CGG) or both cytosines (^m^C^m^CGG) at the corresponding sites. On the other hand, the number of MSAPs present in the *Eco*RI/*Msp*I profile was higher (117 vs 94) and most of the MMS fragments (91.67%) were found associated with *Msp*I digestion. This lower level of variation may be associated with a higher percentage of fully methylated cytosines at CG motif (C^m^CGG) in *Pinus pinea* genome. These results are in agreement with the highest levels of cytosine methylation found in the CG motif observed in both plants and animal genomes [72]. In Arabidopsis it is mainly found in heterochromatic regions with transposable elements and repeats, as well as in genic regions. Cytosine methylation in CNG sequence motif (where N denotes A, T or C; in our case N is C), which is also frequent in plant genomes, is associated with histone modification and involved in small non-coding RNA biogenesis in Arabidopsis [73]. From an adaptive perspective, modification of their methylation status may allow trees to rapidly respond to abrupt changes in environmental conditions as well as to deal with long term responses to more general environmental scenarios [74].

The extent of cytosine methylation at CCGG sites was statistically different among stone pine individuals, ranging from 42.73% to 47.90%. PMS fragments among vegetatively propagated plants obtained from each original tree were used to estimate variability among the 20 trees initially analyzed. Polymorphism levels ranged from 0.46% (Tor 3) to 9.72% (Don 13). This variability may be associated with the developmental stage of the plant or/and differences in their growing environmental conditions. Although all clonally propagated trees shared their chronological stage, methylation variability may be in part associated with differences in their ontological stage, since each ramet derived from a different branch of the corresponding one-year-old mother tree, developing a specific rooting pattern. Additionally, soil heterogeneity among plant pots and micro-environmental variation among plants due to the block design may be associated to some extent with methylation variability.

It is known that MSAP technique could help assessing genetic variability of the analyzed samples since PMI fragments can be associated with mutations on the restriction site in individuals lacking the fragment. However, considering the absence of genetic variation in the species, also supported by the AFLP analysis, PMI-MSAP fragments (5.09% of the total number of MSAPs) detected in trees from five populations of stone pine should be mainly associated with fully methylated ^m^C^m^CGG restriction sites, which are demethylated in those individuals with fragment presence. All these results indicate that epigenetic variability is independent from genetic variability in this species and therefore underscore the potentially important role of the epigenetic variability as an evolutionary mechanism [24], [75].

To acquire a better understanding of the evolutionary implications of this biological process, additional experiments are required to study modification of the cytosine methylation status in response to different environmental conditions (i.e. drought, different atmospheric CO_2_ concentrations, etc.) as well as transgenerational inheritance of these epigenetic marks at both genome - and candidate gene-level.

In relation with genomic resources and from an agronomic point of view, the lack of genetic variation has limited the advance of breeding programs of the species. *P. pinea* is an economic important tree species mainly due to its edible seeds and elite clones for cone production are cultivated in grafted plantations using mainly non-clonal rootstocks [76]. Limitation to identify both, elite tree cuttings and rootstocks, makes more difficult a reliably selection and effective discrimination of materials with a superior capacity for pine nuts yield. The MSAP technique opens an alternative that is worth exploring in order to achieve tree discrimination. In this study we detected a total of 15 PMS that were present or absent in all propagated trees obtained from each original tree (i.e. same profile among ramets from a mother tree but different profiles among different mother trees) that allow to distinguish 14 out of the 20 original trees analyzed. Due to the reduced set of markers in comparison with the number of analyzed samples the epigenetic relationships among the 14 trees were not determined, as indicated by the low bootstrap values obtained at most of the nodes. Additional PMS markers with potential discriminant power could be identified using additional primer combinations. A suitable number of markers can potentially be useful for elite tree identification, supporting stone pine breeding programs with a reliable method to identify improved materials. However, DNA methylation status of cytosines at target CCGG restriction sites from a given organ may vary, as mentioned above, due to plant ontogeny or environmental changes. It is therefore critical to determine the stability of any selected PMS-MSAP marker in different developmental stages and contrasting growing conditions.

Population differentiation for conservation purposes is also a major issue in this species. Different provenances have been identified along the Spanish natural distribution based on environmental characteristics (climatic and geographic) and historical human intervention (fires, clear-cuts, reforestations) but without a genetic structure supporting it [77]. Recently, a set of nuclear microsatellites with medium-low or low polymorphic information content have been identified and used to analyze, in a broad sense, stone pine population structure [45]. Additionally, inter-population variability has been described for growth related phenotypic traits in common garden assays [48], [49]. MSAP analysis offers the opportunity to study a source of variability unexplored to date [78]. Although the low number of individuals per population in this work does not reach the typical approach from population genetic studies, a preliminary analysis showed epigenetic differences among populations. AMOVA and PCA performed over the two Spanish populations represented in this study with a higher number of trees, Tordesillas and Bogarra, showed that MSAP fragments were informative enough to clearly differentiate them, in contrast with the single AFLP pattern shared between all the samples that made it impossible to distinguish both populations. PCA results showed how ramets from each population clustered together along the first component in a two-dimensional scatter plot. In addition, it was possible to identify smaller clusters of ramets corresponding to propagated trees. Even though genome-wide methylation levels were similar among populations, a high percentage of polymorphic MSAPs showed significant epigenetic differentiation between these populations.

Epigenetic variability has been suggested to contribute to the phenotypic plasticity and adaptive potential of individuals and populations and therefore to their evolution [16], [79]. Several studies have suggested that epigenetic variability alone can cause heritable variability in phenotypic traits [5], [14], [19], [27] although its effect on fitness has still to be elucidated. *Pinus pinea* is a genetically depauperated but plastic species. Our results reveal a high level of cytosine methylation in stone pine genome as well as high levels of variation of methylation between the analyzed trees. These results, together with the high levels of phenotypic plasticity observed in the species, may suggest a potential role of cytosine methylation in the regulation of gene expression and variation in phenotypic traits to improve *Pinus pinea* fitness under different environmental conditions. Further analysis of methylation pattern evolution in stone pines subjected to different forecasted environmental conditions, whether isolated events or recurrent stresses, associated with different future scenarios (i.e. water availability, different atmospheric CO_2_ concentrations, temperature, etc.), should be carried out to confirm this hypothesis.

## Supporting Information

Figure S1 **Map showing the natural distribution of** ***Pinus pinea*** **L. and the location of the studied populations.** Map source: EUFORGEN (modified).
(PDF)

Table S1 **Location, climatic characteristics and number of propagated trees and ramets per tree of the studied populations.**
(PDF)

Table S2 **Sequences of adaptors and primers used in MSAP and AFLP assays.**
(PDF)

Table S3 **MSAP markers showing statistically significant epigenetic differentiation among populations.**
(PDF)

Table S4 **Binary matrix codifying the scoring of the MSAP fragment patterns for both primer combinations.** First and second numbers on each cell represent the independent scoring of *Eco*RI/*Hpa*II and *Eco*RI/*Msp*I patterns, respectively. The scoring codes were 1 for fragment presence, 0 for fragment absence, and 9 for missing data.
(XLSX)

## References

[1] Zhang M, Kimatu JN, Xu K, Liu B (2010) DNA cytosine methylation in plant development. J Genet Genomics 37: 1–12 Available: http://www.ncbi.nlm.nih.gov/pubmed/20171573. Accessed 2013 June 24. 20171573 10.1016/S1673-8527(09)60020-5

[2] Raissig MT, Baroux C, Grossniklaus U (2011) Regulation and flexibility of genomic imprinting during seed development. Plant Cell 23: 16–26 Available: http://www.pubmedcentral.nih.gov/articlerender.fcgi?artid=3051244&tool=pmcentrez&rendertype=abstract. Accessed 2013 July 24. 21278124 10.1105/tpc.110.081018 PMC3051244

[3] Tsukahara S, Kobayashi A, Kawabe A, Mathieu O, Miura A, et al (2009) Bursts of retrotransposition reproduced in Arabidopsis. Nature 461: 423–426 Available: http://www.ncbi.nlm.nih.gov/pubmed/19734880. Accessed 2013 July 24. 19734880 10.1038/nature08351

[4] Bucher E, Reinders J, Mirouze M (2012) Epigenetic control of transposon transcription and mobility in Arabidopsis. Curr Opin Plant Biol 15: 503–510 Available: http://www.ncbi.nlm.nih.gov/pubmed/22940592. Accessed 2013 July 24. 22940592 10.1016/j.pbi.2012.08.006

[5] Verhoeven KJF, van Dijk PJ, Biere A (2010) Changes in genomic methylation patterns during the formation of triploid asexual dandelion lineages. Mol Ecol 19: 315–324 Available: http://www.ncbi.nlm.nih.gov/pubmed/20015141. Accessed 2012 Oct 27. 20015141 10.1111/j.1365-294X.2009.04460.x

[6] Li A, Hu BQ, Xue ZY, Chen L, Wang WX, et al (2011) DNA methylation in genomes of several annual herbaceous and woody perennial plants of varying ploidy as detected by MSAP. Plant Mol Biol Rep 29: 784–793 Available: http://www.springerlink.com/index/10.1007/s11105-010-0280-3. Accessed 2014 Jan 22. 24415835 10.1007/s11105-010-0280-3 PMC3881574

[7] Richards EJ (2006) Inherited epigenetic variation–revisiting soft inheritance. Nat Rev Genet 7: 395–401 Available: http://www.ncbi.nlm.nih.gov/pubmed/16534512. Accessed 2014 Jan 20. 16534512 10.1038/nrg1834

[8] Richards CL, Bossdorf O, Verhoeven KJF (2010) Understanding natural epigenetic variation. New Phytol 187: 562–564 Available: http://www.ncbi.nlm.nih.gov/pubmed/20659249. 20659249 10.1111/j.1469-8137.2010.03369.x

[9] Bräutigam K, Vining KJ, Lafon-Placette C, Fossdal CG, Mirouze M, et al (2013) Epigenetic regulation of adaptive responses of forest tree species to the environment. Ecol Evol 3: 399–415 Available: http://doi.wiley.com/10.1002/ece3.461. Accessed Accessed 2014 Jan 20. 23467802 10.1002/ece3.461 PMC3586649

[10] Gourcilleau D, Bogeat-Triboulot MB, Thiec D, Lafon-Placette C, Delaunay A, et al (2010) DNA methylation and histone acetylation: genotypic variations in hybrid poplars, impact of water deficit and relationships with productivity. Ann For Sci 67: 208p1–208p10 Available: http://link.springer.com/10.1051/forest/2009101. Accessed 2013 July 24.

[11] Raj S, Bräutigam K, Hamanishi ET, Wilkins O, Thomas BR, et al (2011) Clone history shapes *Populus* drought responses. Proc Natl Acad Sci U S A 108: 12521–12526 Available: http://www.pubmedcentral.nih.gov/articlerender.fcgi?artid=3145742&tool=pmcentrez&rendertype=abstract. Accessed 2013 June 3. 21746919 10.1073/pnas.1103341108 PMC3145742

[12] Lira-Medeiros CF, Parisod C, Fernandes RA, Mata CS, Cardoso MA, et al (2010) Epigenetic variation in mangrove plants occurring in contrasting natural environment. PLoS One 5: e10326 Available: http://www.pubmedcentral.nih.gov/articlerender.fcgi?artid=2859934&tool=pmcentrez&rendertype=abstract. Accessed 2013 May 29. 20436669 10.1371/journal.pone.0010326 PMC2859934

[13] Karan R, DeLeon T, Biradar H, Subudhi PK (2012) Salt stress induced variation in DNA methylation pattern and its influence on gene expression in contrasting rice genotypes. PLoS One 7: e40203 Available: http://www.pubmedcentral.nih.gov/articlerender.fcgi?artid=3386172&tool=pmcentrez&rendertype=abstract. Accessed 2013 June 24. 22761959 10.1371/journal.pone.0040203 PMC3386172

[14] Scoville AG, Barnett LL, Bodbyl-Roels S, Kelly JK, Hileman LC (2011) Differential regulation of a MYB transcription factor is correlated with transgenerational epigenetic inheritance of trichome density in *Mimulus guttatus* . New Phytol 191: 251–263 Available: http://www.pubmedcentral.nih.gov/articlerender.fcgi?artid=3107365&tool=pmcentrez&rendertype=abstract. Accessed 2013 July 24. 21352232 10.1111/j.1469-8137.2011.03656.x PMC3107365

[15] Herrera CM, Bazaga P (2011) Untangling individual variation in natural populations: ecological, genetic and epigenetic correlates of long-term inequality in herbivory. Mol Ecol 20: 1675–1688 Available: http://www.ncbi.nlm.nih.gov/pubmed/21466603. Accessed 2013 June 3. 21466603 10.1111/j.1365-294X.2011.05026.x

[16] Chinnusamy V, Zhu JK (2009) Epigenetic regulation of stress responses in plants. Curr Opin Plant Biol 12: 133–139. Available: http://www.pubmedcentral.nih.gov/articlerender.fcgi?artid=3139470&tool=pmcentrez&rendertype=abstract. 2013 July 24. 10.1016/j.pbi.2008.12.006 PMC3139470 19179104

[17] Mirouze M, Paszkowski J (2011) Epigenetic contribution to stress adaptation in plants. Curr Opin Plant Biol 14: 267–274 Available: http://www.ncbi.nlm.nih.gov/pubmed/21450514. Accessed 2014 Jan 22. 21450514 10.1016/j.pbi.2011.03.004

[18] Zhang YY, Fischer M, Colot V, Bossdorf O (2013) Epigenetic variation creates potential for evolution of plant phenotypic plasticity. New Phytol 197: 314–322 Available: http://doi.wiley.com/10.1111/nph.12010. Accessed 2014 Jan 22. 23121242 10.1111/nph.12010

[19] Cubas P, Vincent C, Coen E (1999) An epigenetic mutation responsible for natural variation in floral symmetry. Nature 401: 157–161 Available: http://www.ncbi.nlm.nih.gov/pubmed/10490023. Accessed 2013 July 24. 10490023 10.1038/43657

[20] Cervera MT, Ruiz-García L, Martínez-Zapater JM (2002) Analysis of DNA methylation in *Arabidopsis thaliana* based on methylation-sensitive AFLP markers. Mol Genet Genomics 268: 543–552 Available: http://www.ncbi.nlm.nih.gov/pubmed/12471452. Accessed 2013 May 21. 12471452 10.1007/s00438-002-0772-4

[21] Keyte AL, Percifield R, Liu B, Wendel JF (2006) Infraspecific DNA methylation polymorphism in cotton (*Gossypium hirsutum* L.). J Hered 97: 444–450 Available: http://www.ncbi.nlm.nih.gov/pubmed/16987937. Accessed 2013 July 4. 16987937 10.1093/jhered/esl023

[22] Vaughn MW, Tanurdzić M, Lippman Z, Jiang H, Carrasquillo R, et al (2007) Epigenetic natural variation in *Arabidopsis thaliana* . PLoS Biol 5: e174 Available: http://www.pubmedcentral.nih.gov/articlerender.fcgi?artid=1892575&tool=pmcentrez&rendertype=abstract. Accessed 2014 July 24. 17579518 10.1371/journal.pbio.0050174 PMC1892575

[23] Kalisz S, Purugganan MD (2004) Epialleles via DNA methylation: consequences for plant evolution. Trends Ecol Evol 19: 309–314 Available: http://www.ncbi.nlm.nih.gov/pubmed/16701276. Accessed 2013 July 3. 16701276 10.1016/j.tree.2004.03.034

[24] Bossdorf O, Richards CL, Pigliucci M (2008) Epigenetics for ecologists. Ecol Lett 11: 106–115 Available: http://www.ncbi.nlm.nih.gov/pubmed/18021243. Accessed 2014 Jan 20. 18021243 10.1111/j.1461-0248.2007.01130.x

[25] Richards CL, Bossdorf O, Pigliucci M (2010) What role does heritable epigenetic variation play in phenotypic evolution? Bioscience 60: 232–237 Available: http://www.jstor.org/stable/10.1525/bio.2010.60.3.9. Accessed 2013 May 27.

[26] Fraga MF, Ballestar E, Paz MF, Ropero S, Setien F, et al (2005) Epigenetic differences arise during the lifetime of monozygotic twins. Proc Natl Acad Sci U S A 102: 10604–10609 Available: http://www.pubmedcentral.nih.gov/articlerender.fcgi?artid=1174919&tool=pmcentrez&rendertype=abstract. Accessed 2013 May 21. 16009939 10.1073/pnas.0500398102 PMC1174919

[27] Johannes F, Porcher E, Teixeira FK, Saliba-Colombani V, Simon M, et al (2009) Assessing the impact of transgenerational epigenetic variation on complex traits. PLoS Genet 5: e1000530 Available: http://www.pubmedcentral.nih.gov/articlerender.fcgi?artid=2696037&tool=pmcentrez&rendertype=abstract. Accessed 2013 May 31. 19557164 10.1371/journal.pgen.1000530 PMC2696037

[28] Jablonka E, Raz G (2009) Transgenerational epigenetic inheritance: prevalence, mechanisms, and implications for the study of heredity and evolution. Q Rev Biol 84: 131–176 Available: http://www.ncbi.nlm.nih.gov/pubmed/19606595. Accessed 2013 July 24. 19606595 10.1086/598822

[29] Verhoeven KJF, Jansen JJ, van Dijk PJ, Biere A (2010) Stress-induced DNA methylation changes and their heritability in asexual dandelions. New Phytol 185: 1108–1118 Available: http://www.ncbi.nlm.nih.gov/pubmed/20003072. Accessed 2012 Nov 8. 20003072 10.1111/j.1469-8137.2009.03121.x

[30] Vining KJ, Pomraning KR, Wilhelm LJ, Priest HD, Pellegrini M, et al (2012) Dynamic DNA cytosine methylation in the *Populus trichocarpa* genome: tissue-level variation and relationship to gene expression. BMC Genomics 13: 27 Available: http://www.pubmedcentral.nih.gov/articlerender.fcgi?artid=3298464&tool=pmcentrez&rendertype=abstract. Accessed 2013 Jan 10. 22251412 10.1186/1471-2164-13-27 PMC3298464

[31] Lafon-Placette C, Faivre-Rampant P, Delaunay A, Street N, Brignolas F, et al (2013) Methylome of DNase I sensitive chromatin in *Populus trichocarpa* shoot apical meristematic cells: a simplified approach revealing characteristics of gene-body DNA methylation in open chromatin state. New Phytol 197: 416–430 Available: http://www.ncbi.nlm.nih.gov/pubmed/23253333. Accessed 2014 Jan 22. 23253333 10.1111/nph.12026

[32] Rohde A, Junttila O (2008) Remembrances of an embryo: long-term effects on phenology traits in spruce. New Phytol 177: 2–5 Available: http://www.ncbi.nlm.nih.gov/pubmed/18078467. Accessed 2013 June 22. 18078467 10.1111/j.1469-8137.2007.02319.x

[33] Schulman E (1958) Bristlecone pine, oldest known living thing. Natl Geogr Mag 113: 354–372.

[34] Gregory TR, Nicol JA, Tamm H, Kullman B, Kullman K, et al (2007) Eukaryotic genome size databases. Nucleic Acids Res 35: D332–D338 Available: http://www.pubmedcentral.nih.gov/articlerender.fcgi?artid=1669731&tool=pmcentrez&rendertype=abstract. Accessed 2013 July 24. 17090588 10.1093/nar/gkl828 PMC1669731

[35] Zonneveld BJM (2012) Conifer genome sizes of 172 species, covering 64 of 67 genera, range from 8 to 72 picogram. Nord J Bot 30: 490–502 Available: http://doi.wiley.com/10.1111/j.1756-1051.2012.01516.x Accessed 2014 Jan 22.

[36] FAO (2010) Global forest resources assessment 2010: main report. Available: www.fao.org/forestry/fra. Accessed 2014 Jan 22.

[37] Fraga MF, Rodríguez R, Cañal MJ (2002) Genomic DNA methylation-demethylation during aging and reinvigoration of *Pinus radiata* . Tree Physiol 22: 813–816 Available: http://www.ncbi.nlm.nih.gov/pubmed/12184986 Accessed 2013 July 24. 12184986 10.1093/treephys/22.11.813

[38] Monteuuis O, Doulbeau S, Verdeil JL (2008) DNA methylation in different origin clonal offspring from a mature *Sequoiadendron giganteum* genotype. Trees 22: 779–784 Available: http://www.springerlink.com/index/10.1007/s00468-008-0238-3 Accessed 2014 Jan 22.

[39] Huang LC, Hsiao LJ, Pu SY, Kuo CI, Huang BL, et al (2012) DNA methylation and genome rearrangement characteristics of phase change in cultured shoots of *Sequoia sempervirens* . Physiol Plant 145: 360–368 Available: http://www.ncbi.nlm.nih.gov/pubmed/22380594 Accessed 2013 July 24. 22380594 10.1111/j.1399-3054.2012.01606.x

[40] Johnsen O, Fossdal CG, Nagy N, Molmann J, DaeHlen OG, et al (2005) Climatic adaptation in Picea abies progenies is affected by the temperature during zygotic embryogenesis and seed maturation. Plant, Cell Environ 28: 1090–1102 Available: http://doi.wiley.com/10.1111/j.1365-3040.2005.01356.x Accessed 2014 June 13.

[41] Yakovlev IA, Fossdal CG, Johnsen Ø (2010) MicroRNAs, the epigenetic memory and climatic adaptation in Norway spruce. New Phytol 187: 1154–1169 Available: http://www.ncbi.nlm.nih.gov/pubmed/20561211 Accessed 2012 Nov 5. 20561211 10.1111/j.1469-8137.2010.03341.x

[42] Yakovlev IA, Lee Y, Rotter B, Olsen JE, Skrøppa T, et al (2014) Temperature-dependent differential transcriptomes during formation of an epigenetic memory in Norway spruce embryogenesis. Tree Genet Genomes 10: 355–366 Available: http://link.springer.com/10.1007/s11295-013-0691-z Accessed 2014 June 2.

[43] Fallour D, Fady B, Lefevre F (1997) Study on isozyme variation in *Pinus pinea* L.: evidence for low polymorphism. Silvae Genet 4: 201–207 Available: http://www.silvaegenetica.de/fileadmin/content/dokument/archiv/silvaegenetica/46_1997/46-4-201.pdf Accessed 2013 January 14.

[44] Vendramin GG, Fady B, González-Martínez SC, Hu FS, Scotti I, et al (2008) Genetically depauperate but widespread: the case of an emblematic Mediterranean pine. Evolution (N Y) 62: 680–688 Available: http://www.ncbi.nlm.nih.gov/pubmed/17983461 Accessed 2012 Nov 16. 10.1111/j.1558-5646.2007.00294.x 17983461

[45] Pinzauti F, Sebastiani F, Budde KB, Fady B, González-Martínez SC, et al (2012) Nuclear microsatellites for *Pinus pinea* (Pinaceae), a genetically depauperate tree, and their transferability to *P. halepensis* . Am J Bot 99: e362–e365 Available: http://www.ncbi.nlm.nih.gov/pubmed/22935358 Accessed 2014 Jan 22. 22935358 10.3732/ajb.1200064

[46] Mutke S, Gordo J, Gil L (2005) Cone yield characterization of a stone pine (*Pinus pinea* L.) clone bank. Silvae Genet 54: 189–197 Available: http://sauerlaender-verlag.com/fileadmin/content/dokument/archiv/silvaegenetica/54_2005/54-4-5-189.pdf Accessed 2013 Jan 14.

[47] Chambel MR, Climent J, Alía R (2007) Divergence among species and populations of Mediterranean pines in biomass allocation of seedlings grown under two watering regimes. Ann For Sci 64: 87–97 Available: http://www.afs-journal.org/articles/forest/abs/2007/01/f7010/f7010.html Accessed 2014 Jan 22.

[48] Carrasquinho I, Gonçalves E (2013) Genetic variability among *Pinus pinea* L. provenances for survival and growth traits in Portugal. Tree Genet Genomes 9: 855–866 Available: http://link.springer.com/10.1007/s11295-013-0603-2 Accessed 2013 May 31.

[49] Mutke S, Gordo J, Khouja M, Fady B (2013) Low genetic and high environmental diversity at adaptive traits in *Pinus pinea* from provenance tests in France and Spain. Options Méditerranées: 73–79. Available: http://sostenible.palencia.uva.es/document/gfs/publicaciones/Articulos/Mutke 2013 Opt Medit Provenances.pdf. Accessed 2013 July 25.

[50] Sánchez-Gómez D, Velasco-Conde T, Cano-Martín FJ, Ángeles Guevara M, Teresa Cervera M, et al (2011) Inter-clonal variation in functional traits in response to drought for a genetically homogeneous Mediterranean conifer. Environ Exp Bot 70: 104–109 Available: http://linkinghub.elsevier.com/retrieve/pii/S0098847210001590 Accessed 2014 Jan 22.

[51] Dellaporta SL, Wood J, Hicks JB (1983) A plant DNA minipreparation: Version II. Plant Mol Biol Report 1: 19–21 Available: http://link.springer.com/10.1007/BF02712670 Accessed 2014 Jan 22.

[52] Cervera MT, Cabezas JA, Sancha JC, Martínez de Toda F, Martínez-Zapater JM (1998) Application of AFLPs to the characterization of grapevine *Vitis vinifera* L. genetic resources. A case study with accessions from Rioja (Spain). Theor Appl Genet 97: 51–59 Available: http://www.springerlink.com/openurl.asp?genre=article&id=doi:10.1007/s001220050866 Accessed 2014 Jan 22.

[53] Vos P, Hogers R, Bleeker M, Reijans M, Lee T van de, et al (1995) AFLP: a new technique for DNA fingerprinting. Nucleic Acids Res 23: 4407–4414 Available: http://www.ncbi.nlm.nih.gov/pubmed/10206688 Accessed 2014 Jan 22. 7501463 10.1093/nar/23.21.4407 PMC307397

[54] Cervera MT, Remington D, Frigerio JM, Storme V, Ivens B, et al (2000) Improved AFLP analysis of tree species. Can J For Res 30: 1608–1616 Available: http://www.nrcresearchpress.com/doi/abs/10.1139/x00-085 Accessed 2013 Jan 14.

[55] Reyna-López GE, Simpson J, Ruiz-Herrera J (1997) Differences in DNA methylation patterns are detectable during the dimorphic transition of fungi by amplification of restriction polymorphisms. Mol Gen Genet 253: 703–710 Available: http://www.ncbi.nlm.nih.gov/pubmed/9079881 Accessed 2013 July 24. 9079881 10.1007/s004380050374

[56] Butkus V, Petrauskiene L, Maneliene Z, Klimašauskas S, Laučys V, et al (1987) Cleavage of methylated CCCGGG sequences containing either N4-methylcytosine or 5-methytcytosine with Mspl, Hpall, Smal, Xmal and Cfr9I restriction endonudeases. Nucleic Acids Res 15: 7091–7102 Available: http://nar.oxfordjournals.org/cgi/doi/10.1093/nar/15.17.7091 Accessed 2014 Jan 22. 2821492 10.1093/nar/15.17.7091 PMC306195

[57] McClelland M, Nelson M, Raschke E (1994) Effect of site-specific modification on restriction endonucleases and DNA modification methyltransferases. Nucleic Acids Res 22: 3640–3659 Available: http://nar.oxfordjournals.org/content/22/17/3640.short Accessed 2013 July 24. 7937074 10.1093/nar/22.17.3640 PMC308336

[58] StatSoft (2004) STATISTICA (data analysis software system). Available: www.statsoft.com.

[59] Sneath PHA, Sokal RR (1973) Numerical taxonomy. The principles and practice of numerical classification. H. WF, editor San Francisco.

[60] Mougel C, Thioulouse J, Perrière G, Nesme X (2002) A mathematical method for determining genome divergence and species delineation using AFLP. Int J Syst Evol Microbiol 52: 573–586 Available: http://ijs.sgmjournals.org/content/52/2/573.full.pdf Accessed 2014 June 16. 11931171 10.1099/00207713-52-2-573

[61] Felsenstein J (2005) PHYLIP (Phylogeny Inference Package) version 3.6. Distributed by the author. Department of Genome Sciences, University of Washington, Seattle

[62] Tamura K, Stecher G, Peterson D, Filipski A, Kumar S (2013) MEGA6: Molecular Evolutionary Genetics Analysis Version 6.0. Mol Biol Evol 30: 2725–2729. 24132122 10.1093/molbev/mst197 PMC3840312

[63] Excoffier L, Smouse PE, Quattro JM (1992) Analysis of molecular variance inferred from metric distances among DNA haplotypes: application to human mitochondrial DNA restriction data. Genetics 131: 479–491 Available: http://www.pubmedcentral.nih.gov/articlerender.fcgi?artid=1205020&tool=pmcentrez&rendertype=abstract Accessed 2014 Jan 22. 1644282 10.1093/genetics/131.2.479 PMC1205020

[64] Excoffier L, Lischer HEL (2010) Arlequin suite ver 3.5: a new series of programs to perform population genetics analyses under Linux and Windows. Mol Ecol Resour 10: 564–567 Available: http://www.ncbi.nlm.nih.gov/pubmed/21565059 Accessed 2014 Jan 20. 21565059 10.1111/j.1755-0998.2010.02847.x

[65] Herrera CM, Bazaga P (2010) Epigenetic differentiation and relationship to adaptive genetic divergence in discrete populations of the violet *Viola cazorlensis* . New Phytol 187: 867–876 Available: http://www.ncbi.nlm.nih.gov/pubmed/20497347 Accessed 2013 July 24. 20497347 10.1111/j.1469-8137.2010.03298.x

[66] Salmon A, Clotault J, Jenczewski E, Chable V, Manzanares-Dauleux MJ (2008) Brassica oleracea displays a high level of DNA methylation polymorphism. Plant Sci 174: 61–70 Available: http://linkinghub.elsevier.com/retrieve/pii/S0168945207002701 Accessed 2014 Jan 21.

[67] Marfil CF, Camadro EL, Masuelli RW (2009) Phenotypic instability and epigenetic variability in a diploid potato of hybrid origin, *Solanum ruiz-lealii* . BMC Plant Biol 9: 21 Available: http://www.pubmedcentral.nih.gov/articlerender.fcgi?artid=2656509&tool=pmcentrez&rendertype=abstract Accessed 2014 Jan 22. 19232108 10.1186/1471-2229-9-21 PMC2656509

[68] Nystedt B, Street NR, Wetterbom A, Zuccolo A, Lin YC, et al (2013) The Norway spruce genome sequence and conifer genome evolution. Nature Advance On. Available: http://www.nature.com/doifinder/10.1038/nature12211. Accessed 2013 May 22. 10.1038/nature12211 23698360

[69] Lisch D, Bennetzen JL (2011) Transposable element origins of epigenetic gene regulation. Curr Opin Plant Biol 14: 156–161 Available: http://www.ncbi.nlm.nih.gov/pubmed/21444239 Accessed 2012 May 16. 21444239 10.1016/j.pbi.2011.01.003

[70] Lee JT (2012) Epigenetic regulation by long noncoding RNAs. Science (80-) 338: 1435–1439 Available: http://www.ncbi.nlm.nih.gov/pubmed/23239728 Accessed 2014 Feb 22. 10.1126/science.1231776 23239728

[71] Latzel V, Allan E, Bortolini Silveira A, Colot V, Fischer M, et al (2013) Epigenetic diversity increases the productivity and stability of plant populations. Nat Commun 4: 2875 Available: http://www.ncbi.nlm.nih.gov/pubmed/24285012 Accessed 2014 Jan 21. 24285012 10.1038/ncomms3875

[72] Stroud H, Greenberg MVC, Feng S, Bernatavichute YV, Jacobsen SE (2013) Comprehensive analysis of silencing mutants reveals complex regulation of the *Arabidopsis* methylome. Cell 152: 352–364 Available: http://www.pubmedcentral.nih.gov/articlerender.fcgi?artid=3597350&tool=pmcentrez&rendertype=abstract Accessed 2014 Mar 19. 23313553 10.1016/j.cell.2012.10.054 PMC3597350

[73] Stroud H, Do T, Du J, Zhong X, Feng S, et al (2014) Non-CG methylation patterns shape the epigenetic landscape in Arabidopsis. Nat Struct Mol Biol 21: 64–72 Available: http://dx.doi.org/10.1038/nsmb.2735 Accessed 2014 Mar 19. 24336224 10.1038/nsmb.2735 PMC4103798

[74] Richards EJ (2011) Natural epigenetic variation in plant species: a view from the field. Curr Opin Plant Biol 14: 204–209 Available: http://www.ncbi.nlm.nih.gov/pubmed/21478048 Accessed 2012 June 26. 21478048 10.1016/j.pbi.2011.03.009

[75] Richards CL, Verhoeven KJF, Bossdorf O (2012) Plant genome diversity volume 1. Wendel JF, Greilhuber J, Dolezel J, Leitch IJ, editors Vienna: Springer Vienna. Available: http://www.springerlink.com/index/10.1007/978-3-7091-1130-7. Accessed 2014 Jan 22.

[76] Mutke S, Gordo J, Gil L (2000) The Stone Pine (*Pinus pinea* L.) breeding programme in Castile-Leon (central Spain). FAO Nucis-Newsletter 9: 50–55.

[77] Prada MA, Gordo J, De Miguel J, Mutke S, Catalán-Bachiller G, et al. (1997) Regiones de Procedencia. Pinus pinea. Nacionales P, editor Madrid.

[78] Li Y, Shan X, Liu X, Hu L, Guo W, et al (2008) Utility of the methylation-sensitive amplified polymorphism (MSAP) marker for detection of DNA methylation polymorphism and epigenetic population structure in a wild barley species (*Hordeum brevisubulatum*). Ecol Res 23: 927–930 Available: http://www.springerlink.com/index/10.1007/s11284-007-0459-8 Accessed 2012 Nov 17.

[79] Nicotra a B, Atkin OK, Bonser SP, Davidson AM, Finnegan EJ, et al (2010) Plant phenotypic plasticity in a changing climate. Trends Plant Sci 15: 684–692 Available: http://www.ncbi.nlm.nih.gov/pubmed/20970368 Accessed 2013 July 24. 20970368 10.1016/j.tplants.2010.09.008

