# Supplementary figures and images for: Epigenetic Variability in the Genetically Uniform Forest Tree Species Pinus pinea L
Source: PLoS One. 2014 Aug 1;9(8):e103145. doi: 10.1371/journal.pone.0103145 (PMC4118849; doi:10.1371/journal.pone.0103145)

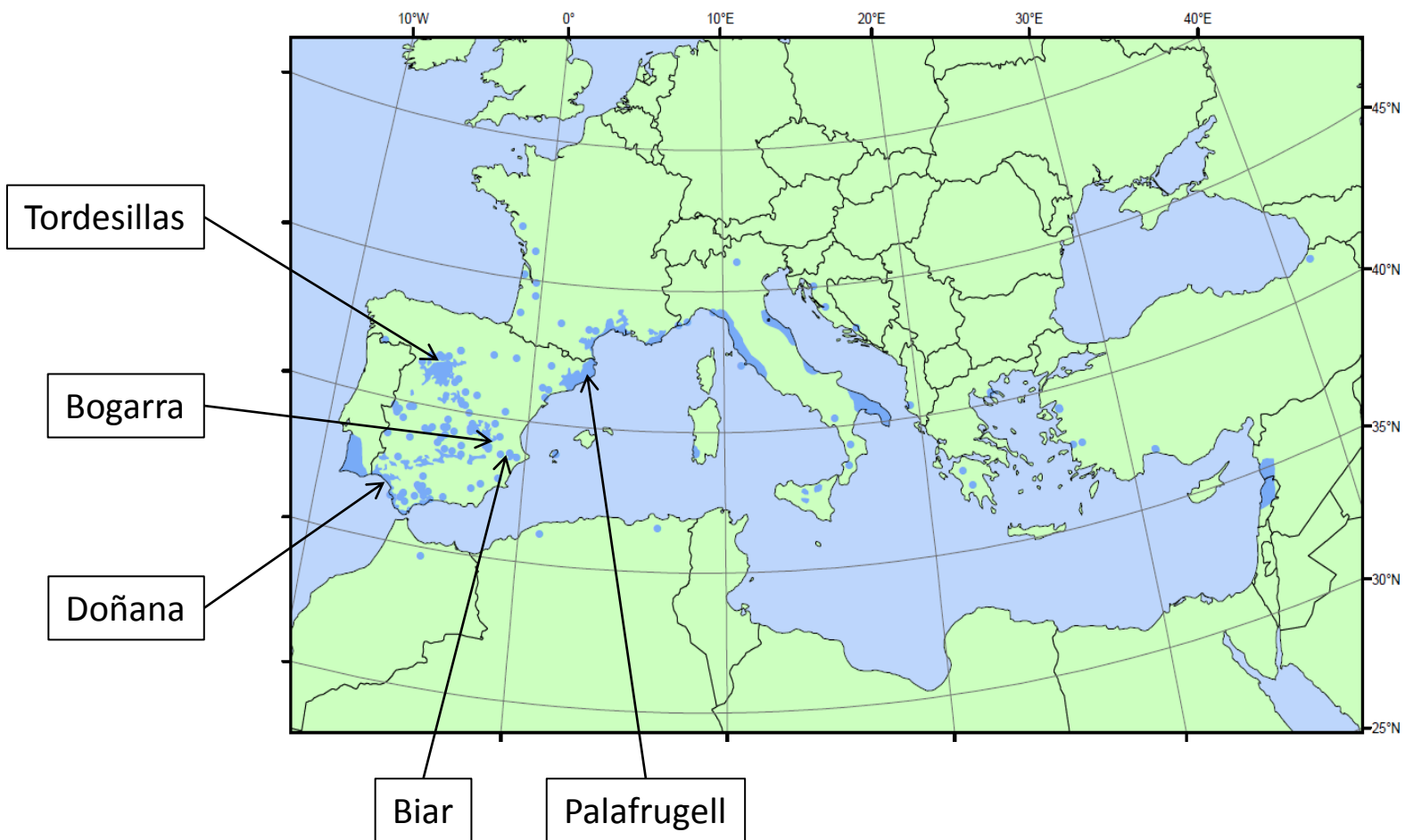

Supplement: Figure S1 — Map showing the natural distribution of Pinus pinea L. and the location of the studied populations. Map source: EUFORGEN (modified). (PDF) [file pone.0103145.s001.pdf]
