# Supplementary material for: Epigenetic Variability in the Genetically Uniform Forest Tree Species Pinus pinea L
Source: PLoS One. 2014 Aug 1;9(8):e103145. doi: 10.1371/journal.pone.0103145 (PMC4118849; doi:10.1371/journal.pone.0103145)
Supplement: Table S1 — Location, climatic characteristics and number of propagated trees and ramets per tree of the studied populations. (PDF) [file pone.0103145.s002.pdf]

Table S1. Location, climatic characteristics and number of propagated trees and ramets per tree of the studied populations.

Population	Coordinates	Altitude	Precipitation	Number of Clones	Number of trees	Number of ramets per tree	Propagated tree code
Tordesillas	41°30’N;	680	452	35	8	3	Tor 3
						6	Tor 7
						3	Tor 12
						4	Tor 13
						4	Tor 24
						6	Tor 25
						5	Tor 27
						4	Tor 29
Bogarra	38°32’N;	800	430	24	5	3	Bor 13
						6	Bor 14
						6	Bor 18
						3	Bor 20
						6	Bor 21
Doñana	36°55’N;	20	631	16	3	5	Don 10
						5	Don 13
						6	Don 15
Biar	38°38’N;	900	359	10	2	3	Bi 23
						7	Bi 37
Palafrugell	41°57’N;	100	710	10	2	5	Pal 19
						5	Pal 27
