# Supplementary material for: Epigenetic Variability in the Genetically Uniform Forest Tree Species Pinus pinea L
Source: PLoS One. 2014 Aug 1;9(8):e103145. doi: 10.1371/journal.pone.0103145 (PMC4118849; doi:10.1371/journal.pone.0103145)
Supplement: Table S2 — Sequences of adaptors and primers used in MSAP and AFLP assays. (PDF) [file pone.0103145.s003.pdf]

Table S2. Sequences of adaptors and primers used in MSAP and AFLP assays.

Sequence type	Assay step	Related endonuclease	Sequence
Adaptors	Ligation	*Eco*RI	5’CTCGTAGACTGCGTACC / 5’AATTGGTACGCAGTC
		*Hpa*II/*Msp*I	5’GACGATGAGTCTCGAT / 5’CGATCGAGACTCAT
		*Mse*I	5’GACGATGAGTCCTGAG / 5’TACTCAGGACTCAT
Primer combination	Pre-amplification	*Eco*RI	5’GACTGCGTACCAATTC-A
		*Hpa*II/*Msp*I	5’GATGAGTCTCGATCGG-A
		*Mse*I	5’ATGAGTCCTGAGTAA-C
	Selective Amplification	*Eco*RI	5’GACTGCGTACCAATTC-AAC/ACA/ACT/ACG/ATC/ACC/CCA
		*Hpa*II/*Msp*I	5’GATGAGTCTCGATCGG-AAT/ACT/ATC
		*Mse*I	5’ATGAGTCCTGAGTAA- CCA
