# Supplementary material for: Epigenetic Variability in the Genetically Uniform Forest Tree Species Pinus pinea L
Source: PLoS One. 2014 Aug 1;9(8):e103145. doi: 10.1371/journal.pone.0103145 (PMC4118849; doi:10.1371/journal.pone.0103145)
Supplement: Table S3 — MSAP markers showing statistically significant epigenetic differentiation among populations. (PDF) [file pone.0103145.s004.pdf]

Table S3. **MSAP markers showing statistically significant epigenetic differentiation among populations.**

MSAP marker id.	Fixation index (F_{ST}))	p-value
7	0,8686	<0,0001
14	0,7738	<0,0001
19	1	<0,0001
20	1	<0,0001
21	0,3833	0,0068
24	0,7655	<0,0001
35	0,5324	0,0108
46	0,4593	0,0166
49	1	<0,0001
51	0,8472	<0,0001
52	0,4790	<0,0001
76	0,6399	<0,0001
78	0,5348	0,0010
82	0,3668	0,0176
89	0,4412	0,0020
91	1	<0,0001
94	0,8867	<0,0001
98	0,3513	0,0352
105	1	<0,0001
115	0,3431	0,0420
122	1	<0,0001
125	0,8443	<0,0001
132	1	<0,0001
138	1	<0,0001
139	1	<0,0001
144	1	<0,0001
149	0,8173	0,0010
151	1	<0,0001
154	1	<0,0001
161	0,4477	0,0284
171	0,9229	<0,0001
176	0,8842	<0,0001
185	0,8012	<0,0001
189	0,8548	<0,0001
190	0,8185	<0,0001
191	0,3582	0,0499
203	1	0,0010
204	1	<0,0001
205	0,6779	0,0010
211	0,8662	<0,0001
217	0,5296	<0,0001
222	0,4014	0,0059
224	1	<0,0001
240	1	<0,0001
241	0,3064	0,0166
251	0,3906	0,0020
253	1	<0,0001
255	1	<0,0001
264	0,4739	0,0020
265	0,8509	<0,0001
266	1	<0,0001
270	1	<0,0001
